# What is the concordance between parent- and education professional-reported adaptive functioning in autistic children using the VABS-II?

**DOI:** 10.1007/s10803-022-05602-2

**Published:** 2022-05-17

**Authors:** Heather L. Moore, Ann Le Couteur, Tony Charman, Jonathan Green, Jeremy R Parr, Victoria Grahame

**Affiliations:** 1 School of Psychology, 4.28, Dame Margaret Barbour Building, Wallace Street, Newcastle upon Tyne, NE2 4DR England, UK; 2 Population Health Sciences Institute, Newcastle University, Sir James Spence Institute, Royal Victoria Infirmary, Level 3, Queen Victoria Road, NE1 4LP Newcastle upon Tyne, UK; 3 Department of Psychology, Institute of Psychiatry, Psychology & Neuroscience, King’s College London, Box PO77, Henry Wellcome Building, De Crespigny Park, Denmark Hill, SE5 8AF London, UK; 4 Division of Neuroscience and Experimental Psychology, University of Manchester, PACT-G Trial Office, Room 3.312, Jean McFarlane Building, Oxford Road, M13 9PL Manchester, UK; 5 Royal Manchester Children’s Hospital, Manchester University NHS Foundation Trust, Oxford Road, M13 9WL Manchester, UK; 6 Cumbria, Northumberland, Tyne and Wear NHS Foundation Trust, Walkergate Park, Benfield Rd, NE6 4QD Newcastle upon Tyne, UK

**Keywords:** Autism, Adaptive functioning, Concordance, Parent-report, Education Professional-/Teacher-Report

## Abstract

Adaptive functioning of autistic children is traditionally measured through informant-report, often from parents. Behaviour varies across settings though, and context-specific reports should be considered. Limited and inconsistent results show low parent-education professional concordance, but no research has yet explored item level response variation. We investigated Vineland Adaptive Behaviour Scales-II concordance using 233 lower ability autistic children from the PACT-G sample. Domain and item level agreement was low, but better on objectively measured behaviours. Higher child nonverbal ability improved concordance. Where disagreements occurred, education professionals identified emergent skills more and parents were more likely to rate present/absent. Parents and education professionals view the adaptive abilities of autistic children differently and both should be considered when developing personalised interventions and support.

Autistic people commonly experience difficulties with adaptive functioning (Maskey et al., 2012). Adaptive functioning involves the practical, everyday skills that an individual needs to meet the demands of their environment, including the ability to communicate and interact with others, access education and complete everyday living tasks. These skills are essential for a number of real-world outcomes such as educational attainment, likelihood of independent living, and reliance on support services (De Bildt et al., 2005; Farley et al., 2009; Taylor & Henninger, 2015) and longitudinal follow up of autistic adults indicates low rates of independent living, employment, friendships and romantic relationships (Magiati et al., 2014; Lord et al., 2020; Zimmerman et al., 2018). Therefore, it is important to understand adaptive abilities of autistic individuals and the long-term outcomes related to these skills, in order to better map out personalised support and improve long term outcomes.

However, in the context of autism, measurement of adaptive functioning has proven challenging for two main reasons. Firstly, autistic children may lack insight into their abilities, may struggle to communicate about their behaviours, and may not demonstrate their skills when assessed directly. This has been addressed by utilising informant-report for adaptive abilities in autistic children. Informants (e.g. parents, teachers) have observed the child’s skills in daily life (Merrell, 2000), and have the added advantage of being able to report less frequent but potentially important behaviours. A commonly used tool is the Vineland Adaptive Behaviour Scales (VABS; (Sparrow et al., 1984a, b, 2005), an informant-report measure that includes domains in Communication, Daily Living Skills (DLS), Socialisation, and Motor Skills, as well as an Adaptive Behaviour Composite (ABC) score that subsumes all domains. Using parent-report, research has identified a broad profile with relative strengths in Motor Skills and weaknesses in Socialisation or Communication (Mouga et al., 2015; Paul et al., 2014; Ventola et al., 2014; Matthews et al., 2015; Nevill et al., 2017; Yang et al., 2016).

The second challenge of measurement in all areas of autism is the strongly context-dependent nature of behaviour, and difficulties with generalising learning between contexts (Carruthers et al., 2020). Autistic children often vary in their behaviour over time and across contexts, such as between home and school, and different expectations may be placed on children in these different environments (Ozonoff et al., 2005; McDonald et al., 2016; De Los Reyes, 2011; Achenbach, 2011; Kanne et al., 2009). Very little research has investigated education professional report of adaptive functioning in autistic children, but a similar profile of relative strengths in Motor Skills was recently identified using education professional-report (Moore et al., 2021). However, the profile of abilities across VABS-II domains was consistent in this research, in contrast to the relative weaknesses in Socialisation or Communication found in the parent literature. Despite this variation in reported profiles, the same child factors influenced reporting by education professionals (namely, negative associations with chronological age, parent-reported autism severity and teacher-rated child behaviour, as well as positive associations with nonverbal ability and language ability) as can be seen in the parent literature (e.g. Di Rezze et al., 2019; Farmer et al., 2018; Nevill et al., 2017; Paynter et al., 2018).

The variation in the information available to informants may lead to differences in reports of adaptive functioning, complicating our ability to understand the adaptive abilities of autistic children. Understanding and interpretation of items, as well as familiarity with the child and frequency of interactions with them may also impact on reporting of adaptive abilities (Achenbach, 2011). To date, only four studies have investigated concordance between parent- and teacher-reported adaptive functioning and have shown varying results (Dickson et al., 2018; Lane et al., 2013; McDonald et al., 2016; Jordan et al., 2019), although differences exist in sample characteristics between the studies.

When comparing overall scores between parents and teachers, a small study by Lane et al., (2013) found no difference using the VABS-II with 28 34–71 month olds, but a subsample of parents rated their children as having significantly better performance than teachers when using the adaptive skills component of the Behaviour Assessment System for Children-2 (BASC-2; (Reynolds & Kamphaus, 2004). A larger study by Dickson et al., (2018) also showed consistently higher parent than teacher ratings on the VABS-II with 246 3–12 year olds (mean cognitive standard score: 68.8, SD: 20.8). Conversely, in two higher functioning samples (IQ > 70), McDonald et al., (2016) and Jordan et al., (2019) both found higher teacher than parent ratings using the BASC-2 and Adaptive Behavior Assessment System-3 (ABAS-3; (Harrison & Oakland, 2015), respectively (McDonald et al.: 118 6–11 year olds; Jordan et al.:103 6–12 year olds). Investigating agreement between parent and teacher scores, Lane and colleagues and Dickson and colleagues found medium to large correlations, whereas McDonald et al. and Jordan et al. found small to medium correlations (Lane et al.: VABS-II ranged from 0.43 to 0.73, though Socialisation subscale scores were not significant in isolation, BASC-2: 0.41, although Adaptability subscale was nonsignificant in isolation; Dickson et al.: 0.44-0.66; McDonald et al.: 0.10-0.29; Jordan et al.: 0.27-0.48). Additionally, McDonald et al. and Jordan et al. identified poor-good intra-class correlations (ICC; McDonald et al.: 0.18-0.43; Jordan et al.: 0.41-0.65). Both also used Bland-Altman plots (plus regression) to determine whether systematic differences emerged across the range of scores, and McDonald et al. found greater parent-teacher difference as the parent-teacher mean score increased on their adaptive skills composite score. Three studies explored predictors of congruence, and while Dickson et al., (2018) identified cognitive ability as a significant predictor for the Social domain, neither McDonald et al., (2016) nor Jordan et al., (2019) found significant correlations with any measured parent or child factors.

Combined, these studies show variable concordance, with differences in whether parents or teachers rated child behaviour higher, agreement between raters ranging from small to large, and variation in whether any identified rater or child factors influenced the level of agreement. It may be that this relates to the specific measure, or to the ability level of the children. Less research has explored concordance between parents and teachers of lower ability autistic children, although Dickson’s research had a wider ability range than the studies conducted by McDonald et al. and Jordan et al. and showed a different pattern of results. Indeed, Voelker et al., (2000) found that parents of non-autistic children with low IQ rated them as more advanced than their teachers. In addition, no study to date has examined whether parents and education professionals are reporting the same behaviours. While domain scores may be similar, these are constructed from total scores across multiple subsections, designed to measure different behaviours, and not all items are consistent across parent and teacher forms. Given the context-specific behaviour presented by autistic individuals, it may be that informants are reporting different behaviours entirely, or different capabilities on the same behaviours, which subsequently combine to affect domain-level agreement. Research by Voelker et al., (2000) suggested different reporting patterns where disagreement existed, with parents tending towards more extreme categories in their rankings than teachers (e.g. present/absent vs. emergent/sometimes present; i.e. abilities coming into being). To our knowledge, no research to date has investigated item level agreement between different raters using the VABS-II in autism. Detailed exploration and comparison of item level responses on the parent and teacher VABS-II would provide an opportunity to more fully understand adaptive functioning in each context.

In order to address this gap in the literature, we investigated concordance between parent- and education professional-reported adaptive abilities in a sample of pre- and primary school aged autistic children with lower nonverbal ability than previously considered. We used the baseline characterisation data of the Paediatric Autism Communication Trial-Generalised (PACT-G) trial (see Green et al., 2018), for details of the trial protocol) for this work. Please note that when referring to measures and associated scores, we use ‘teacher’, which is the label used by these measures, but when referring to informants, we use ‘education professional’, to encompass the broader range of staff involved in this research. Our first aim was to replicate current studies to explore VABS-II ABC and domain concordance between parents and education professionals in the PACT-G sample, by (1) Comparing mean scores, and looking at ICCs and Bland-Altman plots (plus regression); and (2) Exploring child factors that may influence reporting and therefore the level of disagreement between parents and education professionals. Our second aim was to extend the knowledge base by considering to what extent concordance exists at the item level, and if disparities do exist; on which items and why. Given the limited and variable literature base, it is difficult to form directional hypotheses. However, we do anticipate low levels of concordance between parents and education professionals at both the ABC/domain level and the item level.

## Methods

### Participants

In this study, we used the baseline characterisation data from PACT-G (Green et al., 2018), a multisite, randomised controlled trial social communication intervention for autistic children. A sample of 248 children, aged between 2 and 11 years, in South London, Greater Manchester and the North-East of England were recruited via referral from local clinical and educational services between November 2016 and April 2018. Eligibility criteria included: a clinical autism diagnosis, confirmed using the Autism Diagnostic Observation Schedule-2nd Edition (ADOS-2; Lord et al., 2012) cut-off scores for ‘Autism Spectrum Disorder’ and the Social Communication Lifetime Questionnaire (SCQ; Rutter et al., 2003) scores of ≥ 12 (for children < 5 years) or ≥ 15 (for children ≥ 5 years); a nonverbal age-equivalent of > 12 months, measured using the Visual Reception (VR) and Fine Motor (FM) subscales of the Mullen Scales of Early Learning (MSEL; Mullen 1995), or the Special Nonverbal Composite Score on the British Ability Scales-School Age (BAS; Elliott & Smith 2011); and language abilities between P3 and P8 on the English curriculum^1^ for children ≥ 5 years. Children with epilepsy were included if controlled by medication. Children/parents with significant hearing/visual impairments were excluded, as were parents with severe learning disability or psychiatric disorder. Parents were required to have enough spoken and written English to participate in PACT-G assessments and intervention. Child characteristics and parent and education provision information for the whole sample are presented in Table 1. A favourable ethical opinion was obtained from the North West-Greater Manchester Central Research Ethics Committee (REF: 15/NW/0912) and parents provided informed, written consent before taking part in PACT-G. In addition, the child’s education provider was also required to agree to participate in the study.

**Table 1:** Demographic characteristics of PACT-G sample (N = 233)

	N (%)	Mean	SD	Min	Max
Child CA (Months) – Parent Assessment	233 (100)	61.49	21.89	28	131
Child CA (Months) – Education Professional Assessment	233 (100)	62.74	21.81	36	132
Child Gender					
Female	49 (21.03)				
Male	184 (78.97)				
Child Ethnicity					
White-British	128 (54.94)				
White non-British	12 (5.15)				
Mixed/Multiple ethnic backgrounds^a^	21 (9.01)				
Asian/Asian-British	29 (12.45)				
Black/African/Caribbean/Black British	36 (15.45)				
Other ethnic group^b^	7 (3.00)				
Phrase Speech (ADOS-2 Module^c^)					
Module 1	175 (75.11)				
Module 2	58 (24.89)				
Parent					
Mother	216 (92.70)				
Father	16 (6.87)				
Other^d^	1 (0.43)				
Type of School					
Mainstream nursery	86 (36.91)				
Specialist nursery	4 (1.72)				
Mainstream primary school	47 (20.17)				
Mainstream school with SEN/autism resource class	7 (3.00)				
Special school with mixed disabilities	54 (23.18)				
Specialist autism school	34 (14.59)				
Childminder	1 (0.43)				
Type of Education Professional^e^					
Day care provider	23 (9.87)				
General education teacher	158 (67.81)				
Special education teacher	47 (20.17)				
Other^f^	5 (2.15)				

*Note*: (a) Includes mixed white and black Caribbean, white and black African, white and Asian, and any other mixed backgrounds. (b) Includes Arab. (c) ADOS-2 module 1: nonverbal-simple phrases e.g. two words); ADOS-2 module 2: short phrases upwards. (d) Adoptive Mother. (e) Informant-chosen categories from the T-VABS-II; general and special education teachers include a combination of teachers and LSAs. (f) Includes managerial staff and one school’s Communications Champion

### Measures

Characterisation data from the PACT-G sample used in this study included^2^:

### Adaptive Functioning

The VABS-II survey form (P-VABS-II) is an interview-format rating scale used to measure parent/caregiver assessment of a child’s adaptive functioning, from birth to 90 + years. The Teacher rating form of the VABS-II (T-VABS-II; Sparrow et al., 2005) is a questionnaire used to measure teacher assessment of a child’s adaptive ability within an education setting, for those aged 3–21 years. Both P- and T-VABS-II measure four domains: Communication, Daily Living Skills (DLS), Socialisation, and Motor Skills, which, combined, form an overarching Adaptive Behaviour Composite (ABC) score. Communication appraises the child’s use and understanding of spoken and written language. DLS encompasses practical skills the child needs to take care of themselves and respond to the requirements of the home or school community. Socialisation measures how the child interacts with others and uses their free time. Finally, Motor Skills incorporates fine and gross motor movements, coordination, and manipulation of objects. Inter-rater reliability is 0.74 (Sparrow et al., 2005). Chronological age-relevant starting points are employed with both measures, as well as reverse rules to use earlier items if a child does not meet the baseline requirements for their own age. Raw scores were translated into standard scores for each domain, with a mean of 100 and SD of 15.

### Autism Severity

The ADOS-2 (Lord et al., 2012) is a semi structured, play-based assessment of social communication and restricted and repetitive behaviours (RRBs). Modules 1 and 2 were used, covering the age range and verbal ability of the sample, from nonverbal through to phrase speech. A Calibrated Severity Score (CSS) ranging from 1 to 10 was calculated from social affect (SA) and RRB domains, which is standardised in relation to the child’s CA and verbal ability and can be compared across modules. Higher scores indicate more severe autism symptomology.

The SCQ Lifetime (Rutter et al., 2003) is a 40-item, parent-report measure, which requires yes or no responses according to whether or not the child displays particular social communication behaviours and yields a score ranging from 0 to 40. Higher scores indicate more symptoms of autism.

### Non-Verbal Ability

The VR and FM subscales from the MSEL (Mullen, 1995) measure nonverbal ability. We used age equivalent (AE) scores and calculated a nonverbal developmental quotient (NVDQ; see data analysis), because our sample included children outside of the age range to ascertain standard scores (i.e., > 5 years). Higher scores indicate greater nonverbal ability.

### Language Ability

The Receptive (ROWT) and Expressive (EOWT) One Word Picture Vocabulary Test (Martin & Brownell, 2011a, b) are picture-based assessments assessing understanding and use of single words. The child is presented with a picture and asked to name it (EOWT), and presented with a selection of pictures and asked to identify which one the examiner has named (ROWT). We used raw scores to capture performance variation, with higher scores indicating more correct responses, as many participants did not score sufficient correct responses to derive a t-score.

### Procedure

Baseline data were collected prior to randomisation in the PACT-G study. Assessments administered directly with the child were completed either at the research clinic, child’s home and/or educational setting. Parents completed the SCQ during the first session and returned it to the research team. The P-VABS-II was administered via interview with a trained researcher. The T-VABS-II was completed by the education professional, without interviewer support. Education settings chose the most appropriate person to complete questionnaires, based on prior knowledge of the child. An education professional might complete a T-VABS-II for more than one child. Both parents and education staff were given opportunities to ask questions about any items prior to submission.

### Statistical Analysis

This is a secondary analysis of data collected for PACT-G and was powered to meet the primary aims of this study. For these analyses, the N (ranging between 168 and 233 pairs of raters) was sufficient to detect an effect of 0.5 at an α of 0.05, with power > 0.80 (G*Power 3.1.9.7; Faul et al., 2009). Data were prepared and analysed using Stata Version 16 (StataCorp, 2019) and IBM SPSS Statistics Version 25 (IBM Corp, 2017). One family withdrew from the study post-randomisation, and 12 participants were removed from the analyses as they were < 3 years, thus younger than the youngest available normative data for the T-VABS-II. Data were missing if questionnaires were incomplete, e.g. insufficient subscale items completed to calculate domain scores on the VABS-II, or in the case of VABS-II Motor Skills, if participants were older than the CA subscale cut-off of 7 + years. Table 2 shows N values for each variable included in the analyses. Of researcher-administered measures, two participants completed the BAS (Elliott & Smith, 2011) so did not have MSEL NVDQ scores, and it was not possible to complete the ROWT and EOWT with a small number of participants.

**Table 2:** Descriptive statistics for parent and teacher measures of adaptive functioning, as well as measures of autism severity, nonverbal ability, language ability, and strengths and difficulties

	N	Min	Max	Mean	SD
Parent SS					
P-VABS-II ABC	231	40	87	62.13	9.41
P-VABS-II Communication	233	36	97	62.71	14.89
P-VABS-II DLS	233	34	105	63.15	11.71
P-VABS-II Socialisation	233	47	90	63.50	9.21
P-VABS-II Motor skills	198	43	114	71.02	10.64
Teacher SS					
T-VABS-II ABC	223	25	98	58.22	14.56
T-VABS-II Communication	229	27	114	62.01	16.47
T-VABS-II DLS	231	23	108	62.15	15.61
T-VABS-II Socialisation	233	41	93	61.73	11.40
T-VABS-II Motor Skills	191	30	103	67.79	13.86
Parent-Teacher Mean Difference SS					
PT-VABS-II ABC	222	-42	50	4.10	15.11
PT-VABS-II Communication	229	-55	53	0.85	19.28
PT-VABS-II DLS	231	-52	47	1.02	16.94
PT-VABS-II Socialisation	233	-36	39	1.77	13.32
PT-VABS-II Motor Skills	168	-33	50	2.97	14.64
Child Characteristics					
ADOS CSS	233	6	10	7.40	1.26
SCQ Total Score	233	12	36	23.53	5.29
MSEL NVDQ	231	17.09	112.24	48.69	18.38
ROWT Total Score	232	0	80	18.16	21.68
EOWT Total Score	228	0	78	15.44	18.69

Some of the data were not normally distributed; therefore, we used nonparametric equivalents, where possible. To investigate whether differences existed in the standard scores for parents and education professionals, we compared VABS-II ABC scores using a Wilcoxon signed ranks test. We compared domain scores using a mixed ANOVA, with informant as the between subjects variable and VABS-II domain as the within subjects variable. There is no nonparametric equivalent to a mixed ANOVA, but ANOVAs are considered robust to violations of normality.

We also conducted ICCs to explore the level of agreement between parent and education professional ratings on the VABS-II ABC and domain scores. The strength of agreement on the ICCs was established using Cicchetti & Sparrow (1981) guidelines, developed for assessment of adaptive behaviour (< 0.40: Poor; 0.40-0.60: Fair; 0.60-0.75: Good; 0.75 − 1.00: Excellent).

Additionally, Bland-Altman plots (including regression analyses) were produced to investigate whether systematic differences existed between parent and education professional ratings across the range of scores, for VABS-II ABC and domain scores. These were plotted with the VABS-II Difference Scores on the Y axis, and the mean parent-education professional score on the X axis. Education professional scores were taken from parent scores to calculate the difference score, such that a positive score indicated higher parent rating and a negative score indicated higher education professional rating. An average of the respective parent and education professional score was calculated for VABS-II ABC and domain scores to produce the mean parent-education professional score. On each graph, the solid line represents the mean difference score, and 95% CIs are represented by dotted lines above and below the mean score line. As recommended by Bland & Altman (1986), we also ran regression analyses to determine any systematic trends across the range of scores, by regressing the mean parent-education professional score on to the parent- education professional difference score. Regression lines were added to Bland-Altman plots.

Next, we investigated factors which might affect differences in VABS-II ABC and domain scores between parents and education professionals. The VABS-II was administered to parents and education professionals as close together as possible. However, children were significantly older when rated by education professionals than when rated by parents (Z=-11.18, p < .001; ∆Mean: 1.25 months, see Table 1); therefore, we included difference in chronological age (CA Difference) as a potential predictor of VABS-II Difference Scores. As there were no significant differences between MSEL VR and FM AE scores (Z=-0.23, p = .8086; Mean MSEL VR = 27.43 months, Mean MSEL FM = 27.24 months), we used a mean score to calculate the MSEL NVDQ (mean nonverbal mental AE/CA*100). Participants performed significantly better on the ROWT than EOWT (Z=-4.83, p < .001); therefore, we considered these predictors separately, rather than deriving a language quotient. First, we used Spearman’s rank correlations to explore associations between VABS-II Difference Scores on the ABC and other domains, and child characteristics, such as CA Difference, autism severity, nonverbal ability, and language ability. We subsequently undertook a series of multiple linear regression analyses to explore concurrent associations between VABS-II Difference Scores on the ABC and other domains, and child characteristics. Variables for the regression analyses were entered if they were significantly associated with any of the VABS-II Difference Scores.

Finally, we explored item-level agreement in ratings on the VABS-II for parent and education professional ratings. We first extracted all identical and ‘near identical’ items from the measures, allowing differences in wording relating to ‘home’/‘school’/‘classroom’ etc., but where the content was the same and no other differences in meaning arose. This totalled 165 identical /or near identical items (43% of parent items and 74% of teacher items (see Supplementary Table 1 for items). Although many of the identical items were not administered on the parent form, due to discontinue rules, the assumption is that the child would score zero on items after ceiling, making comparison possible on all identical items. Weighted kappa cross-tabulations for non-unique raters were performed using linear weightings, and simes procedure was applied to correct for multiple comparisons. We established the strength of significant weighted kappa cross-tabulations using guidelines from Altman (1991): ≤0.20: Poor; 0.21-0.40: Fair; 0.41-0.60: Moderate; 0.61-0.80: Good; 0.81 − 1.00: Very good. To further understand differences in rating decisions by parents and education professionals, the distribution of ratings across the categories were examined for all nonsignificant items (i.e., those showing disagreement), to determine how parents and education professionals ranked behaviour. For each nonsignificant kappa score, the item was examined to determine which of parents and education professionals used each category more frequently.

## Results

### Comparison of Parent and Education Professional Adaptive Functioning

**Fig. 1:**
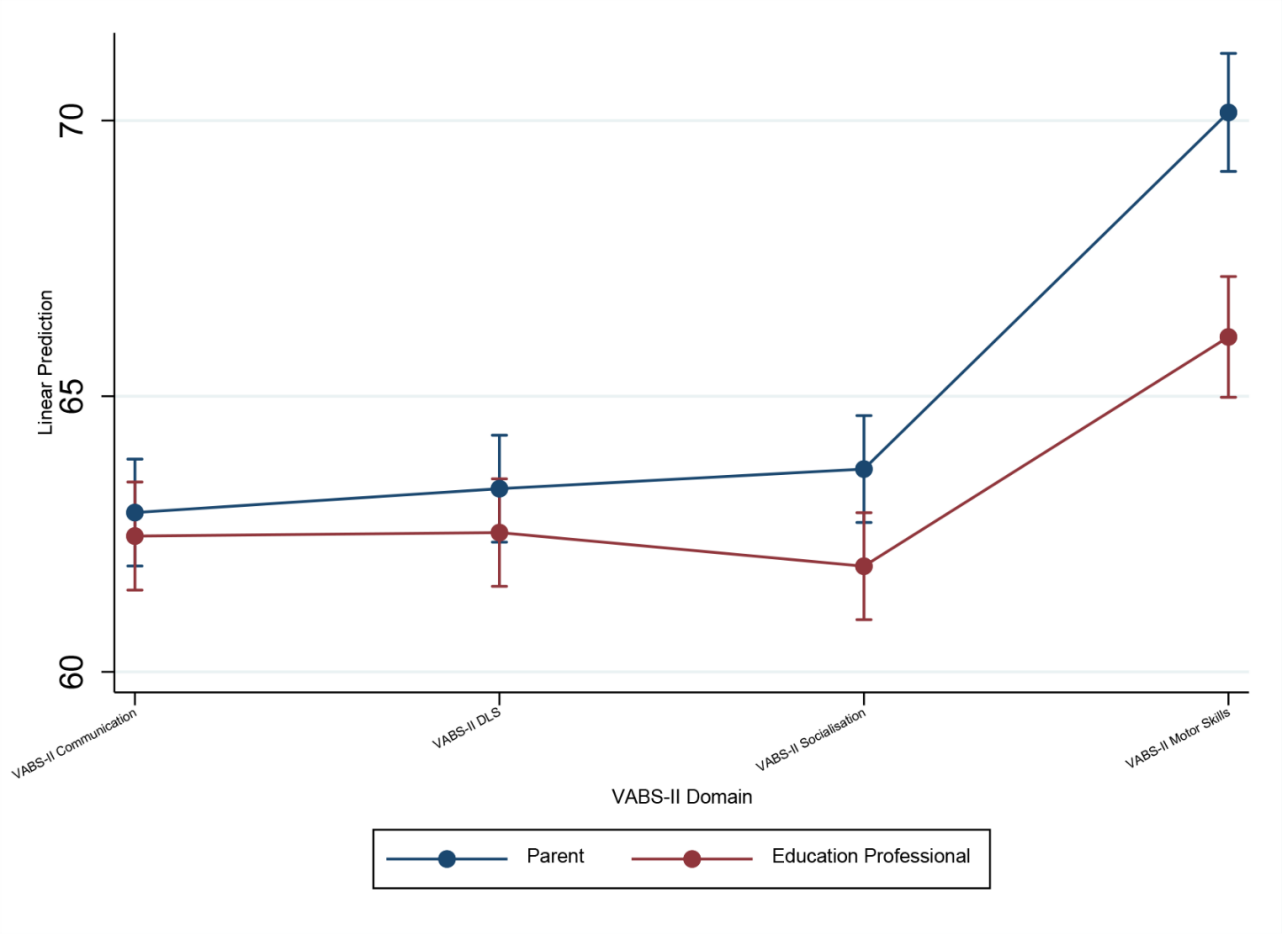
Parent and teacher VABS-II domain scores

Table 2 shows descriptive statistics for P- and T-VAB-II ABC and domain scores. Overall, parents rated their child’s performance significantly higher than education professionals did on VABS-II ABC (N = 222, Z = 4.44, p < .0001). A mixed ANOVA found no significant main effect of informant (F(1, 1309) = 3.97, p = .1018). There was, however, a significant main effect of VABS-II domain (F(3, 1309) = 48.32, p < .0001; Greenhouse-Geisser correction) and a significant Informant by VABS-II domain interaction (F(3, 1309) = 4.69; p = .0039; Greenhouse-Geisser correction). Pairwise comparisons indicated that both parents and education professionals rated VABS-II Motor Skills higher than all other domains (all p < .001), but no other domains differed significantly. As illustrated in Fig. 1, there was no difference in rating between parents and education professionals for VABS-II Communication (N = 229, Z = 1.06, p = .2908, ns) and VABS-II DLS (N = 231, Z = 1.03, p = .3031, ns) domains, whereas parents rated their child’s performance significantly higher than education professionals on VABS-II Socialisation (N = 233, Z=-2.18, p = .0294) and VABS-II Motor Skills (N = 168, Z=-2.77, p = .0054) domains.

### Concordance between Parent and Education Professional Adaptive Functioning

**Fig. 2:**
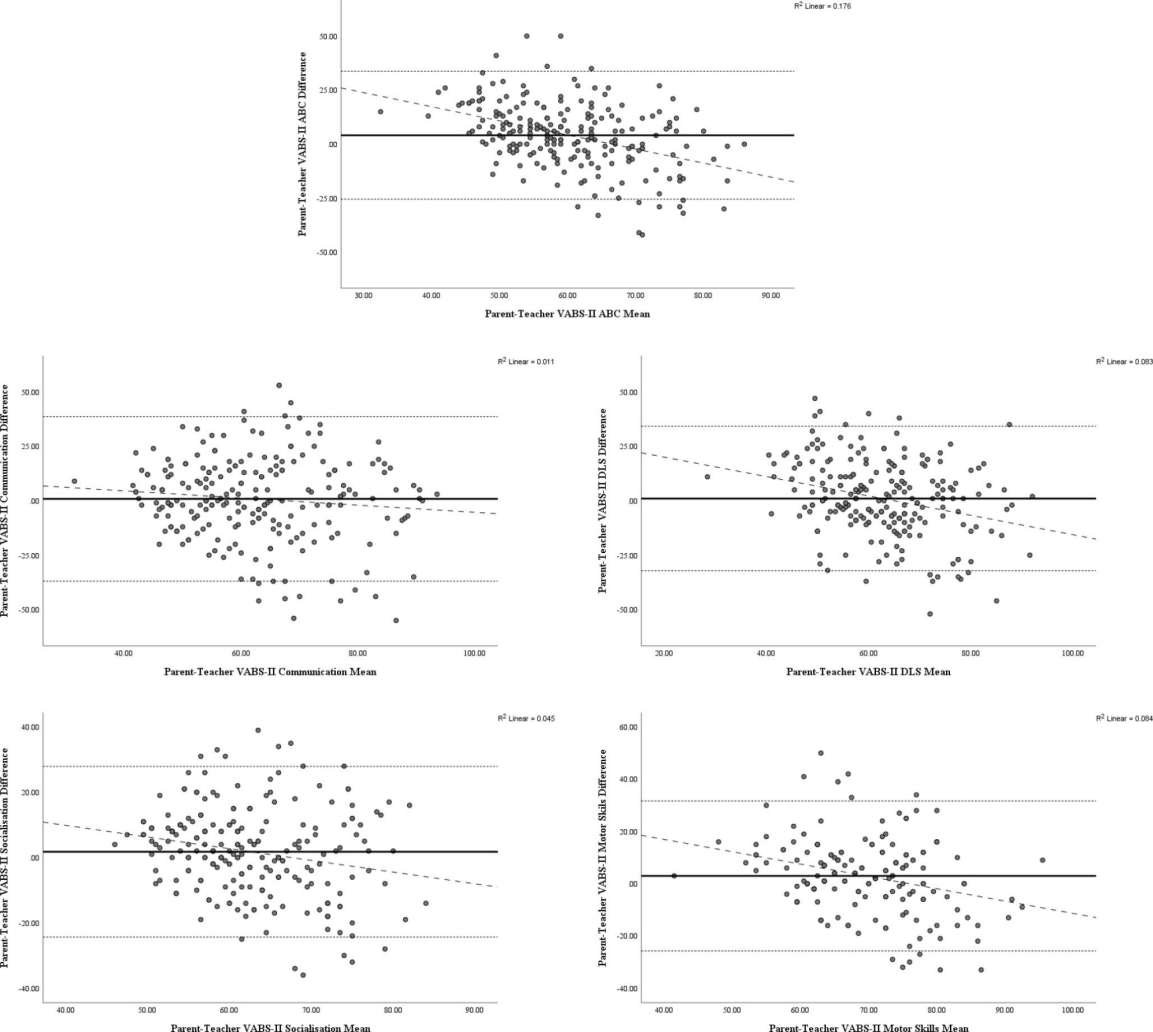
Bland-Altman plots to investigate systematic differences in parent and education professional reporting across the range of scores for VABS-II ABC and domains. *Note*: The solid line represents the mean difference score, dotted lines above and below the mean score line represent 95% CIs, and dashed lines represent regression lines

As can be seen in Table 3, the ICC for inter-rater reliability on the VABS-II ABC and domain scores was poor for all but VABS-II DLS, which demonstrated fair reliability. Figure 2 shows the Bland-Altman plots for VABS-II ABC and domain scores. The range and mean differences in VABS-II SS between parents and education professionals are shown in Table 2. The regression analyses showed a significant negative relationship between the difference scores and means for VABS-II ABC (*B*= -0.65, *t*= -6.85, p < .001), DLS (*B*=-0.45, *t*=-4.54, p < .001), Socialisation (*B*= -0.36, *t*= -3.32, p = .0011), and Motor Skills. VABS-II Communication was not significant (*B*= -0.16, *t*= -1.60, p = .1118).

**Table 3:** ICCs comparing agreement and consistency between parent and education professional ratings for the VABS-II ABC and domain scores

		ICC		
Parent-Teacher Comparison	N	ICC	LLCI	ULCI
VABS-II ABC	222	0.38***	0.19	0.52
VABS-II Communication	229	0.39***	0.21	0.53
VABS-II DLS	231	0.40***	0.22	0.53
VABS-II Socialisation	233	0.29**	0.09	0.45
VABS-II Motor Skills	168	0.33**	0.10	0.50

*Note*: LLCI: Lower level confidence interval; ULCI: Upper level confidence interval; *: Sig < 0.05; **: Sig < 0.01; ***: Sig < 0.001

### Impact of child factors on parent- education Professional Concordance

Table 2 shows VABS-II Difference scores for ABC and domain scores, as well as descriptive statistics for other child characteristics. Significant correlations from bivariate analyses (Supplementary Table 2) guided variable inclusion in regression analyses. Regression analyses for VABS-II ABC and domain difference scores are reported in Table 4. Across all five models, the only significant predictor was MSEL NVDQ, which was negatively associated with difference in scores between parents and education professionals. Variance accounted for by the models ranged from 10 to 35%.

**Table 4:** Regression models for VABS-II adaptive ability difference scores for parents and education professionals, using CA difference, autism severity, nonverbal ability, and language ability

	B (CI)	SE B	β	p	Adjusted r^2^
**VABS-II ABC Difference**					**0.35**
(Constant)	23.11 (13.25, 32.97)	5.00		< 0.001***	
SCQ	0.17 (-0.15, 0.49)	0.16	0.06	0.3058	
MSEL NVDQ	-0.45 (-0.56, -0.35)	0.54	-0.57	< 0.001***	
ROWT	-0.03 (-0.19, 0.13)	0.08	-0.05	0.7005	
EOWT	0.02 (-0.16, 0.20)	0.09	0.03	0.8287	
**VABS-II Communication Difference**					**0.26**
(Constant)	26.07 (19.69, 32.46)	3.24		< 0.001***	
MSEL NVDQ	-0.48 (-0.62, -0.33)	0.07	-0.46	< 0.001***	
ROWT	-0.05 (-0.27, 0.17)	0.11	-0.05	0.6755	
EOWT	-0.06 (-0.30, 0.19)	-0.06	0.28	0.6454	
**VABS-II DLS Difference**					**0.34**
(Constant)	26.77 (15.86, 37.67)	5.53		< 0.001***	
SCQ	0.05 (-0.30, 0.40)	0.18	0.02	0.7827	
MSEL NVDQ	-0.57 (-0.69, -0.45)	0.06	-062.	< 0.001***	
ROWT	0.01 (-0.17, 0.19)	0.09	0.01	0.9456	
EOWT	0.51 -(0.15, 0.25)	0.10	0.06	0.6133	
**VABS-II Socialisation Difference**					**0.14**
(Constant)	8.51 (-1.15, 18.16)	4.90		0.0839	
Child CA	-0.74 (-1.56, 0.08)	0.42	-0.11	0.0782	
SCQ	-0.23 (-0.09, 0.54)	0.16	0.09	0.1570	
MSEL NVDQ	-0.22 (-0.32, -0.11)	0.05	-0.31	< 0.001***	
ROWT	0.06 (-0.10, 0.22)	0.08	0.10	0.4500	
EOWT	-0.12 (-0.31, 0.06)	0.09	-0.18	0.1758	
**VABS-II Motor Skills Difference**					**0.10**
(Constant)	17.48 (10.56, 24.39)	3.50		< 0.001***	
MSEL NVDQ	-0.29 (-0.44, -0.15)	0.08	-0.37	0.0001***	
ROWT	0.05 (-0.08, 0.17)	0.06	0.07	0.4818	

*Note*: ***: Sig < 0.001

### Parent and Education Professional Concordance at the Item Level

Supplementary Table 1 shows the weighted kappa scores and significance (simes correction) for each item. Weighted kappa scores were calculated for 147 of 165 items (89.1%). Scores that were not calculated were often later items in the measures, used for older/more able children, and so lacked sufficient range in scores to determine concordance in our sample. Of the 147 kappa scores, 48 were significant (32.7%), indicating inter-rater agreement. Table 5 shows the percentage with significant inter-rater agreement within each of the 11 subscales that map on to the four domains (using parent subscale categories). Agreement was most frequent for the VABS-II Communication domain, followed by VABS-II DLS domain, and least frequent for the VABS-II Socialisation domain. Within the VABS-II Communication domain, significant inter-rater reliability was most frequent for the Written subscale, and least frequent for the Receptive subscale. Higher frequency agreement within the VABS-II DLS domain came from the Personal subscale. Overall, 30/48 (62.5%) of significant weighted kappas showed poor agreement, and 18/48 (37.5%) of significant weighted kappas showed fair agreement.

**Table 5:** Total number of items and percentage of items with significant kappa weighted crosstabulation scores, for each VABS-II domain and subscale (using parent form subscale categories)

Domain / Subscale		N (N calculated)	No. Significant Kappa Scores (% N / % N Calculated)
VABS-II Communication		55 (44)	28 (50.9 / 63.6)
	Receptive	11	2 (18.2)
	Expressive	25 (21)	14 (56.0 / 66.7)
	Written	19 (12)	12 (63.2 / 100)
VABS-II DLS		25 (24)	10 (40.0 / 41.7)
	Personal	13	7 (53.8)
	Domestic	1	0 (0.0)
	Community	11 (10)	3 (27.3 / 30.0)
VABS-II Socialisation		48 (45)	4 (8.3 / 8.9)
	Interpersonal Relationships	18 (17)	3 (16.7 / 17.6)
	Play and Leisure	16 (15)	1 (6.3 / 6.7)
	Coping Skills	14 (13)	0 (0.0)
VABS-II Motor Skills		37 (34)	6 (16.2 / 17.6)
	Gross Motor Skills	14	3 (21.4)
	Fine Motor Skills	23 (20)	3 (13.0 / 15.0)

*Note*: N: Total number of identical items available for each domain/subscale; N Calculated: Total number of items for which a weighted kappa result was returned. Individual item scores, SE, and simes corrected significance information can be found in Supplementary Table 1

Table 6 shows that where disagreements arose (99/147), education professionals rated behaviour in the ‘sometimes or partially’ category, whereas parents were more likely to rate behaviours as ‘never’ or ‘usually’. To note, frequency of rating ‘never’ was closer for parents and education professionals than the other two categories, but parents chose this ranking more frequently (see Supplementary Table 3 for full cross-tabulations for each item).

**Table 6:** Percentage of VABS-II items on which either parents or education professionals rated children more frequently for each scoring category

	Rater (%)		
VABS-II Scoring Category	Parent	Education Professional	Tie
0 (Never)	72.7	23.2	4.0
1 (Sometimes or Partially)	3.0	94.9	2.0
2 (Usually)	65.7	24.2	10.1

*Note*: Percentages are based on the 99 items for which parents and education professionals disagreed

## Discussion

As far as the authors are aware, this is the first study to investigate the degree of parent and education professional concordance on the VABS-II, a measure of adaptive functioning, at the item level as well as the domain level. Our results show that parents scored their children higher on VABS-II ABC scores, as well as Socialisation and Motor Skills domains, but not Communication or DLS domains. The overall picture was one of poor concordance both at the domain and item level, and systematic differences in ratings across the range of scores (except for VABS-II Communication). Differences at the domain level were greater when child nonverbal ability was lower, but the degree of discrepancy was unaffected by child language or autism severity. Where disagreements occurred at the item level, parents and education professionals frequently chose different response options. However, item level agreement was more frequent for domains/subdomains that measured overt behaviours (e.g. VABS-II Communication, particularly the Written subdomain).

### Parent and Education Professional Agreement

Our study showed higher parent than education professional ratings of adaptive functioning on the VABS-II ABC, and Socialisation and Motor Skills domains, but no difference in ratings on the VABS-II Communication and DLS domains. This is partially consistent with Dickson et al., (2018) and Lane et al., (2013), who found higher parent scores on all domains (on the VABS-II and BASC-2, respectively). However, the literature base is inconsistent; Lane and colleagues also found no difference using the VABS-II, and both McDonald et al., (2016) and Jordan et al., (2019) found higher teacher ratings when using the BASC-2 and ABAS-3, respectively.

Inter-rater reliability between parent and education professionals was poor for all VABS-II ABC and domain scores except DLS, which was fair. Previous research has, again, demonstrated inconsistent findings, with correlations ranging from small to large, and ICCs from poor to good, but with an overall pattern of stronger agreement than seen in the current study (Dickson et al., 2018; Jordan et al., 2019; Lane et al., 2013; McDonald et al., 2016). Our regression analyses indicated systematic differences across the range of scores, with a negative relationship between means and differences for all except VABS-II Communication, which was nonsignificant. Taking these with the Bland-Altman plots, this suggests that at lower mean scores, parents may rate their child higher, whereas as the means increased, education professionals were more likely to rate the child’s performance higher. In contrast, Jordan et al., (2019) found no systematic differences, while McDonald et al., (2016) found greater differences on the BASC-2 ABC as the mean score increased.

### Association between Agreement and Child Characteristics

The discrepancy in all VABS-II scores in our sample was significantly negatively associated with child nonverbal ability, predicting 10–35% of the variance in difference scores, but neither child language nor autism severity were significantly associated with this discrepancy. Thus, as child nonverbal ability increased, the discrepancy between parents and education professionals decreased. This partially corresponds with the results of Dickson et al., (2018), who found significant associations between cognitive ability and parent-teacher congruence on the VABS-II Socialisation domain in their sample. On the other hand, neither Jordan et al., (2019) or McDonald et al., (2016) found significant correlations between difference scores and any child factors.

Differences in ability level of our sample in comparison to samples from previously published research might have contributed to the variation in results. The PACT-G sample has lower ability than other published research, which may lead to differences in perceptions about ability. Indeed, our results align most closely with those of Dickson et al., (2018), whose sample represents a lower ability level than other published research in this area. A higher ability sample may have shown a different pattern of results, with closer agreement and perhaps even education professional scores surpassing those of parents. Our finding that higher child nonverbal ability was associated with reduced discrepancy in VABS-II scores supports this. Further support comes from our Bland-Altman plots and regression analyses, which suggested not only that higher adaptive ability led to closer agreement, but also that parents scored their children higher when the mean adaptive functioning score was lower, but this was reversed when the mean score was higher.

More research is needed to delineate how sample and informant characteristics affect informant report and differences in reporting between multiple informants. Parent education did not affect the degree of concordance on measures of adaptive functioning in previous research (Jordan et al., 2019; McDonald et al., 2016). However, other parent and family factors, such as parent employment status, single parent home, and number of children in the household, are associated with discrepancy in parent and teacher ratings of behavioural difficulties (Cheng et al., 2018), and the impact of these wider characteristics on adaptive functioning congruence in autistic children have not yet been explored. Furthermore, no research has yet included the impact of education professional characteristics (e.g. education level, prior experience with autism) on adaptive functioning concordance. These would benefit from further exploration in future. Another important factor to consider when exploring concordance is the quality of the relationship between the parent and education professional. Minke et al., (2014) found the congruence between parents’ and teachers’ assessment of their relationship quality impacted teacher but not parent scores of social functioning, as well as behaviour problems, but not adaptive skills (specifically measuring adaptability and leadership), in low ability children. It would be interesting to explore whether congruence impacts not only individual parent or teacher scores, but also the degree of agreement between parties.

### Agreement at the Item Level

At the item level, there was significant agreement for around one third of comparable items. However, of these, almost two thirds showed poor agreement, and just over one third presented fair agreement. The highest frequency of item agreement centred on Communication items, whereas the lowest agreement was with Socialisation items, despite specific social communication impairments required for autism diagnosis. Agreement appeared strongest for more objectively identifiable items, such as those from the Written subscale in the Communication domain, compared to the Receptive subscale, which requires a more subjective assessment of understanding for a greater number of items. Again, within the DLS domain, most frequent agreement came from the Personal subscale, which directly measures overt behaviours. Where disagreements arose, parents were more likely to assess their child’s skills on items as either present or absent, whereas education professionals were more likely to rate behaviour as emergent/sometimes present, corresponding to patterns of responding observed by Voelker et al., (2000) in low ability, non-autistic children.

Two key factors might have created meaningful differences in ratings between parents and education professionals. First, discrepancies in informant report may be capturing differences in child behaviour across different contexts (Achenbach, 2011; De Los Reyes, 2013; Alexander et al., 2017). For example, parents might observe a different range of social opportunities with more skilled social partners, who might scaffold the interaction and present a different level of performance than observable by education professionals in a school setting. In line with this, there may be greater opportunities for children to demonstrate their motor skills to parents where a more individualised approach may be possible, routines may be less carefully structured and again, peer play partners may be more skilled. Conversely, communication and DLS might be less influenced by context and expectations placed on the child in home and school settings. In support, De Los Reyes et al., (2013) found that discrepancies increased following training to focus on context-specific behaviours. Very little research has explored associations between informant report and objective observations. One study, comparing parent and teacher report of disruptive behaviour in young children to observations of parent-child and researcher-child interactions, found parent report was specifically associated with parent-child observations, and teacher report with researcher-child observations alone (De Los Reyes et al., 2009). Moreover, the predictive validity of teacher ratings is superior to parents when considering child mental health in an education setting (Aitken et al., 2017). Other studies have identified nonshared environmental rater-specific variance in reports of attention and internalising problems in twins (Bartels et al., 2007; Derks et al., 2006).

The second factor that may influence discrepancies is level of insight (Alexander et al., 2017). Parents and education professionals may apply different thresholds for reporting behaviours (De Los Reyes & Kazdin, 2005; Achenbach, 2011). With their greater training on and experience of child developmental norms and levels in school reporting, education professionals may be better able to distinguish between emerging skills and competence. This can be seen in the variation in preference towards scoring present/absent vs. emergent in parents and education professionals, respectively. Domains with higher agreement (e.g. communication and DLS) may be subsumed by more easily observable items where distinctions between performance are more clearly defined (Achenbach, 2011); e.g., if the child can copy their own first name, or if they are toilet trained during the day. Alternatively, education professionals may have a different sampling base (especially in special schools) for rating decisions, and may be more influenced in their response by comparison of individual skills with other children in the school than parents are. Without extensive comparison of adaptive behaviours of autistic children in both home and school settings, it will not be possible to determine why reports of behaviour differ between parents and education professionals, and whether context, level of insight, or both, play a significant role.

The question becomes whether a combined approach is better at predicting long-term outcomes, and if so, how best to incorporate this information to best effect. Psychopathology research has suggested that using the higher rating (from among different informants), or the rating that endorses a behaviour, increases predictive validity to identify current support needs (e.g. referrals) and longitudinal functional outcomes (e.g. internalising and externalising behaviours, academic, and social functioning; Shemmassian & Lee 2016; Lapalme et al., 2020). Other methods such as triangulating three ratings, and creating a trait score have also demonstrated success over single informant methods (e.g. Makol et al., 2020; Schwab et al., 2020; Styck et al., 2021). While this research demonstrates the value of integrated multi-informant approaches when looking at different areas of psychopathology, it remains to be seen (1) Whether predictive validity of adaptive functioning (and associated) outcomes of autistic children would improve with multi-informant approaches, and (2) What the best method for combining data is, when moving away from diagnosis/referrals. Longitudinal research may help to elucidate the value of different approaches to multi-informant reporting in this area.

It is important to consider the utility of employing multi-informant approaches to understand adaptive functioning in autistic children. Our results indicate low agreement across domains and differences in item level rating thresholds; clearly, parent ratings cannot be assumed to be analogous to education professional ratings, and vice versa. Thus, there is value to gathering information from a range of sources to determine the adaptive abilities of autistic children across contexts (both environmental and person-specific). There is a time cost to collecting information from multiple sources though, so this may best be implemented when both the context and level of insight are relevant in measurement of adaptive ability (Alexander et al., 2017). In research, multi-informant measures are important where goals include generalisability across settings or where interventions are being carried out in more than one setting. Clinically, multi-informant methods may be most effective where healthcare professionals are determining the holistic needs of an autistic child across settings, rather than when difficulties relate to a specific context. Indeed, Decker et al., (2021) recently demonstrated significantly worse psychotherapy outcomes for adolescents where there was greater parent-clinician disagreement about functioning. Such disagreements may lead to disengagement with proposed interventions. Intervention and support planning for adaptive functioning (whether developed by healthcare or educational professionals) should engage with the views of key informants in different settings, to develop context-specific support plans and optimise learning across settings.

### Strengths and Limitations of the current study

One of the major strengths of our study is the large sample of autistic children, with lower ability than previously explored in multi-informant concordance research about adaptive functioning. Nevertheless, we acknowledge some methodological limitations to our measurements. While we used the widely accepted method for calculating child MSEL NVDQ with AE scores, we acknowledge the limitations of ordinal numbers which may represent nonlinear developmental changes across AE scores (see Mervis & Klein-Tasman (2004) for a methodological discussion). We also lack knowledge of education professional characteristics that may potentially affect reporting behaviours. There is an assumption that education professionals have a baseline level of understanding of the behaviours necessary to code each item in the VABS-II; however, these informants differed by education level and teaching experience, as well as autism training and experience, class size and potentially, familiarity with the child. Another potential limitation is that education professionals completed a questionnaire, but researchers interviewed parents for information to determine the rating for each item. Previous research has used questionnaires for both informants, and the interview format may have impacted concordance in this study. It is important to note that results may have been influenced by different floors for standard scores across ages on parent and teacher versions of the VABS-II. However, Voelker et al., (2007) found higher skills estimates on classroom than survey forms when both were completed by teachers, suggesting that our education professional scores may not have been impacted by lower floors. Risk of method variance could be reduced in future by using the same rating form for parents and education professionals (Voelker et al., 2007; Charman et al., 2004).

## Conclusions

In conclusion, these findings have important clinical findings for assessing adaptive functioning of autistic children in different contexts. Parents and education professionals view the adaptive abilities of autistic children differently and both should be considered when gathering information about functioning and developing personalised interventions and support. Additional research is needed to understand why these differences occur, and to explore how best to combine reports for optimal predictive validity about current needs and future outcomes.

**Declarations**.

The Paediatric Autism Communication Trial-Generalised (PACT-G) is funded by the National Institute of Health Research (NIHR) and Medical Research Council [Efficacy and Mechanism Evaluation Programme (13/119/18)].

HLM, ALC, JRP, and VG have no relevant financial or non-financial interests to disclose. TC has served as a paid consultant to F. Hoffmann-La Roche Ltd. and Servier; and has received royalties from Sage Publications and Guilford Publications. JG is a National Institute for Health Research (NIHR) Senior Investigator. The views expressed are those of the authors and not necessarily those of the NHS, the NIHR or the Department of Health and Social Care. JG receives Director’s fees from a not-for-profit PACT training company IMPACT (CiC 10,902,031).

A favourable ethical opinion was obtained from the North West-Greater Manchester Central Research Ethics Committee (REF: 15/NW/0912). Parents provided informed, written consent, and the child’s education provider agreed to participate.

Access to PACT-G data will be available in due course subject to consideration by the PACT-G Consortium and current NIHR guidance.

All authors contributed to the study conception and design. Material preparation and data collection were performed by HLM. Analysis was performed by HLM. The first draft of the manuscript was written by HLM and all authors commented on previous versions of the manuscript. All authors read and approved the final manuscript.

## Footnotes

1 In England, P scales describe targets for children aged 5–16 years with special educational needs. P8 was taken to represent a language age-equivalent of approximately 4 years in a typically developing child.

2 Data from selected measures were used for the purpose of this study. For complete baseline characterisation data of the PACT-G sample, please refer to Green et al. (2018).
